# Impact of SARS-CoV-2 Lockdown on Anthropometric Parameters in Children 11/12 Years Old

**DOI:** 10.3390/nu13114174

**Published:** 2021-11-21

**Authors:** Oliver Ramos-Álvarez, Víctor Arufe-Giráldez, David Cantarero-Prieto, Alba Ibáñez-García

**Affiliations:** 1 Education Faculty, Elviña University Campus, University of A Coruña, 15008 A Coruña, Spain; 2 Education Faculty, Physical Education and Sport Area, Interfacultative Building, University of Cantabria, 52 De los Castros Ave, 39005 Santander, Spain; 3 Research Unit of School Sports, Physical Education and Psychomotricity (UNIDEF), Specific Didactics Department, Research and Diagnostic Methods in Education, Education Faculty, Elviña University Campus, University of A Coruña, 15008 A Coruña, Spain; v.arufe@udc.es; 4 Department of Economics, University of Cantabria, 62A De los Castros Ave, 39006 Santander, Spain; david.cantarero@unican.es; 5 Health Economics and Health Services Management Research Group, IDIVAL Valdecilla, 39011 Santander, Spain; alba.ibanez@unican.es; 6 Education Faculty, Personality, Psychological Assessment and Treatments Area, Interfacultative Building, University of Cantabria, 52 De los Castros Ave, 39005 Santander, Spain

**Keywords:** SARS-CoV-2, lockdown, physical activity, children, anthropometric parameters

## Abstract

Background: During the great lockdown in Spain due to SARS-CoV-2, in between the months of March and June 2020, dietary and physical activity habits were modified in children. The aim of the study was to determine the impact of the lockdown on anthropometric parameters in children aged 11/12 years. Methods: Fifty Spanish children aged 11/12 years (M = 11.40; SD = 0.50) participated: 33 (66%) boys and 17 (34%) girls. For data collection, we used an anthropometry kit; the Alpha-Fitness Battery, a validated instrument to assess dietary intake, habits and practices; and an ad hoc survey to collect sociodemographic and other data under investigation. Results: There were significant differences in the anthropometric parameters measured and in the results obtained for Body Mass Index (BMI) and body fat percentage pre- and post-lockdown in both boys and girls (*p* < 0.05). No significant differences were found in waist-circumference measurements (*p* > 0.05). Conclusions: There is evidence of a significant impact of the SARS-CoV-2 lockdown on anthropometric parameters in boys and girls aged 11/12 years.

## 1. Introduction

On 31 December 2019, a new type of coronavirus appeared in the city of Wuhan (China) that infects and replicates in the pneumocytes and macrophages of the lung parenchyma, where the ACE-2 cell receptor resides, causing symptoms of fever, dry cough, lymphopenia, dyspnoea and pneumonia in its severe form that can lead to the patient’s death. This virus has been called SARS-CoV-2 or COVID-19 and only one month after its emergence in the Chinese city of Wuhan, the World Health Organization (WHO) declared it an international emergency disease [1,2]. The appearance of SARS-CoV-2 on the international scene, and in Spain in particular, led to the population being confined to their homes to prevent the spread of the virus. In Spain, 98 days of home lockdown were decreed between 15 March and 21 June 2020 for all people who did not have essential jobs, limiting by Royal Decree the movement of the population for essential activities [3]. This great lockdown led to the closure of all educational establishments at all levels of education, from nursery schools to universities. Likewise, all schools and sports facilities were closed, so that the practice of any physical activity was reduced to the home. This unprecedented event, not only in Spain but also in other countries (e.g., Italy, France or Portugal), changed certain habits in the child and adolescent population. Habits related to the time dedicated to daily physical activity and the time spent using new technologies were modified [2,4].

Some indirect studies have determined by means of surveys that the school and adolescent population with obesity has changed their habits with the appearance of SARS-CoV-2 and has manifested itself in important health problems. The main health consequences were an increase in weight, an increase in comorbidities, an increase in BMI, statistically significant (*p* < 0.05) parameters in laboratory results related to metabolic diseases and a general worsening of their health, regardless of SARS-CoV-2 status [5]. In line with these studies, the present research aims to show that home lockdown and school closures due to the health crisis caused by SARS-CoV-2 have led to a significant decrease in physical activity, contributing to an increase in childhood and adolescent obesity, as well as to the appearance or worsening of their comorbidities.

At present, there are still few studies related to SARS-CoV-2 in pediatric patients (0–18 years). In Spain, the seroprevalence rate in the pediatric population is estimated to be 3.9% and 3.6% in Cantabria where the research sample is from [6]. These seroprevalence data were obtained by stratified sampling by province and municipality size between 27 April and 11 May 2020, dates falling within the period of the great Spanish confinement. In the case of while the data in the pediatric population worldwide is estimated to be 1.56% [7]. With regard to the incidence of clinical disease of SARS-CoV-2, the World Health Organization (WHO) publishes data worldwide, without a breakdown by age, so there are no official data from this institution for the pediatric population, which is also the case for data on the mortality rate due to COVID-19 [8]. However, different epidemiological agencies in countries and regions of the world [8] have begun to provide these data in a disaggregated manner, as is the case of the Spanish Ministry of Health, which presents data by age in its monitoring reports on the disease [9].

In terms of risk factors for severe SARS-CoV-2 disease, age, arterial hypertension, cardiac or pulmonary problems, diabetes, cancer or obesity seem to be the most predisposing to severe disease according to the information provided by the WHO [10,11,12]. Childhood obesity and overweight can have repercussions on children’s health throughout their lives, hence the importance of working on the prevention of this disease and the acquisition of healthy habits. However, given the prevalence of childhood overweight and obesity in Spain, it may be one of the most important risk factors in the pediatric population [13]. For this reason, changes in anthropometric parameters, as well as in dietary habits and physical activity, may accentuate this risk factor. In addition, children infected with COVID-19 have been identified as having significantly lower vitamin D levels than the pediatric population not infected with COVID-19 [14], a vitamin that is essential for muscle and bone function related to physical activity [15].

In this context, the main factors in the incidence of overweight and obesity in children are sedentary lifestyles and excessive time spent using technology. These factors have led to 85% of school-age girls and 78% of school-age boys not performing the minimum minutes of daily physical activity established by the WHO for their age range [16]. These factors, together with inadequate eating habits, in which ultra-processed foods abound, lead to an obesogenic environment in children that causes an increase in overweight and obesity rates [4,17].

The WHO establishes at least 60 min of moderate to vigorous physical activity (MVPA) per day for children, and the time that exceeds this amount will result in greater health benefits for the child. This institution also recommends that children aged 5–12 can be exposed to screens and new technologies in between 60 and 90 min per day [16,18]. However, the school population is far from complying with these WHO recommendations. Between the ages of 9 and 15 years, there is a greater sedentary time and a decrease in physical activity outside the school context, especially in girls and in schoolchildren who are already overweight or obese compared to schoolchildren with Body Mass Index (BMI) values in the normal range [19].

These low levels of physical activity among schoolchildren and excessive time spent using new technologies, known as the “technological sedentary lifestyle” [4,20], aggravate health problems due to childhood overweight and obesity and cause other health problems, such as isolation of children, poor social relations, sleep disorders, endocrine, musculoskeletal and/or cardiovascular problems [17].

The WHO points out that it is in developed countries where overweight and obesity are the most worrying and most prevalent metabolic problem among the population, calling it the “epidemic of the 21st century” [21]. The prevalence of overweight in children between 6 and 9 years old is 23.3%, and it is 17.3% in the case of obesity, of which 4.2% refers to severe obesity. The data show an upward trend during the school years [22]. The main consequences are an increased risk of mortality, worsening anthropometric parameters and poor physical fitness [23]. They can also lead to cardiovascular problems, diabetes or non-communicable diseases in children and adolescents [22,24,25].

Prior to the appearance of COVID-19 in Spain, a study was already underway, and programmed data were collected, which, together with a collection of post-confinement data, have made the present research possible. For these reasons, the main aim of this study was to find out the impact that the large-scale lockdown due to SARS-CoV-2 had on anthropometric parameters in the population of 11/12-year-old children. The relationships between these anthropometric parameters, dietary habits and sociodemographic data (e.g., type of housing, place of residence or time spent in physical activity) were also studied. In this research, the anthropometric parameters established in the Body Dimension of the Alpha-Fitness Battery [26].

## 2. Materials and Methods

### 2.1. Study Design

In order to carry out this research, a longitudinal observational study was conducted [27]. The dependent variables of this research were the anthropometric parameters of weight, height, waist circumference, triceps skinfold and subscapular skinfold. These parameters were taken according to the Alpha-Fitness Battery, a validated field test for the assessment of health-related physical fitness in children and adolescents [26]. The independent variables were obtained from a validated instrument to assess food consumption, habits and practices in children aged 8–11 [28] and from an ad hoc survey for parents to collect sociodemographic data on the family and data on the different variables under study.

### 2.2. Participants

The sample selected for this research was of a non-probabilistic sample of convenience nature from a primary school in Cantabria (Spain).

A total of 55 children in the sixth year of Primary Education at a school were invited to participate, of which 5 students did not wish to take part in the research for various reasons or because they did not have the informed consent of their parents or legal guardians. The sample finally consisted of 50 schoolchildren between 11 and 12 years of age (M = 11.40; SD = 0.50), 33 (66%) boys and 17 (34%) girls. Fifty-six percent of the sample resided in an urban setting, while 38% resided in a semi-urban or residential setting and 6% in a rural setting.

### 2.3. Tools

The Alpha-Fitness Battery, a validated field test for the assessment of health-related physical fitness in children and adolescents, was used for data collection [26]. The Alpha-Fitness Battery consists of five dimensions: Dimension 1, Tanner Stage (3 items); Dimension 2, Body Composition (5 items); Dimension 3, Musculoskeletal Capacity (3 items); Dimension 4, Motor Capacity (1 item); and Dimension 5, Aerobic Capacity (1 item). For this study, Dimension 2 was used, consisting of five items referring to Body Composition and its reference values [29]: weight (kg), height (cm), waist circumference (cm), triceps skinfold (mm) and sub-scapular skinfold (mm). Measurements were taken by a Holtain mechanical plicometer with a measuring range of 0 to 48 mm and a constant pressure of 10 g/m^2^ mm. A Garmin Index S2 scale, a Seca 2016 measuring rod with a range of 10–230 cm and 1 mm division and a CESCORF anthropometric measuring tape 6 mm wide and 2 m long were also used. The procedure used for the measurement of the anthropometric variables was the one established in the International Standards for Anthropometric Assessment of the International Society for the Advancement of Kineanthropometry [30]. BMI and body fat percentage values were obtained by measuring these variables. For their calculation, the formula BMI = kg/height in m^2^ was used, while for the calculation of body fat percentage, the equations of Slaughter et al. (1988) were followed [31].

For the collection of information on eating habits, a validated instrument was used to assess food consumption, habits and practices in children aged 8–11 [28]. This instrument is made up of 42 items distributed in five sections: frequency of food consumption, cooking skills, eating habits, expenditure on food in the school environment and knowledge.

An ad hoc survey consisting of 50 items was also used for parents or legal guardians. It was used to collect sociodemographic data on the family, time spent on physical activity before and during lockdown, time spent on sedentary activities and the use of new technologies and emotional aspects during lockdown.

### 2.4. Procedure

**Figure 1:**
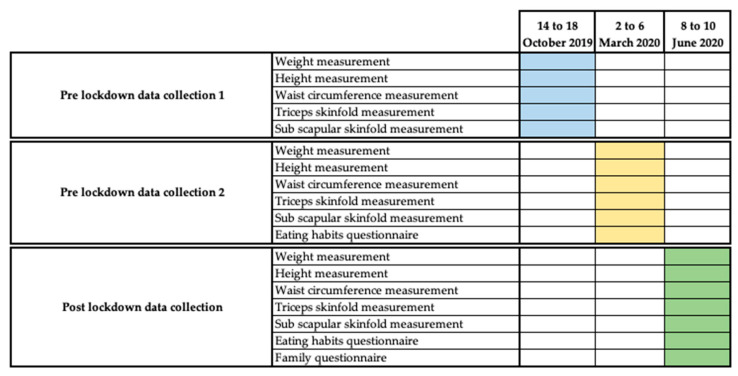
Data-collection schedule. Questionnaires and averages.

The aim of this research is to find out the impact that lockdown due to SARS-CoV-2 had on anthropometric parameters in the 11/12-year-old population. The research has its origins in a broader investigation that aimed to carry out a comparative analysis between two groups of sixth year primary school children with respect to anthropometric parameters; physical condition; psychological and emotional aspects, such as anxiety; and academic results. Data collection was scheduled to take place at three points during the academic year. The first two data collections were carried out as planned in physical-education classes [32] during the weeks of 14 October 2019 and 2 March 2020 (Figure 1).

However, the third data collection, due to the outbreak of SARS-CoV-2 in Spain, could not be carried out at the scheduled time. This outbreak led to a major house lockdown in Spain from 15 March 2020, decreed by the Spanish State by means of a State of Alarm. During this lockdown all educational centers in Spain are closed indefinitely [3]. This is why the third data collection was carried out immediately after the end of this major lockdown and during the de-escalation period from 28 May 2020 (Figure 1). In order to guarantee the sanitary measures established by the Spanish government for the prevention of SARS-CoV-2 infection, the sample was convened in groups of 6 children at different times and in an open-air space.

Both the initial study and its modification followed the processes established at the administrative and ethical level for research with schoolchildren: authorization by the educational centers and the Inspection Service, an informative meeting with the families or legal guardians who constituted the research sample and changes in the established schedule. At this meeting, the objectives and process of the research (data collection, analysis techniques and use of the data collected), the confidentiality of the participants, the voluntary nature of the study and the possibility for their children to leave the study at any time they wished without the need to justify their withdrawal from the study were explained. All this information was given to the families in writing, together with the informed consent form.

### 2.5. Statistical Analysis

SPSS statistical software (SPSS v.26, IBM Corporation, New York, NY, USA) was used to perform all the statistical analyses of the study. A descriptive analysis of the main variables under investigation was carried out, as well as normality tests of quantitative variables for the testing of hypotheses. The Kolmogorov–Smirnov statistic (*n* > 50) was used for the normality analyses of the whole sample, while the Shapiro–Wilk statistic (*n* < 50) was used for the normality tests by sex. When the *p*-value of the normality tests was significant (*p* < 0.05), the hypothesis that the variable does not have a normal distribution was accepted.

For the hypothesis testing, different tests of independence have been used depending on the nature of the variables and certain assumptions that must be fulfilled in order to apply them. Whether the quantitative variable is normally distributed in the different categories of the qualitative variable (parametric tests) or whether it is non-normally distributed in the different categories of the qualitative variable (non-parametric tests), the type of test will also depend on whether the categorical or qualitative variable has two or more than two categories. For parametric tests, when the categorical variable has two categories, the Student’s *t*-test was used, and if it has three or more categories, the comparison of means was carried out through the analysis of variance ANOVA. In the non-parametric analyses, when the categorical variable has two categories, the Mann–Whitney U test was used, and if it has three or more groups, the Kruskal–Wallis test was used. The Student’s *t*-test for paired samples was also used to check if there is a statistically significant difference (*p* < 0.05) between the data obtained pre-lockdown and post-lockdown of the study variables, in case the assumption of normality was met, if there was no normality, the non-parametric Wilcoxon rank test was used. For independence between qualitative variables, the chi-square test of independence was used.

Finally, to test for correlation or association between quantitative variables, two tests were used depending on whether their distribution is normal or not. When the distribution of both variables is normal, Pearson correlation was used; otherwise, Spearman correlation was used.

### 2.6. Ethical Aspects

The ethical and deontological principles established by the American Psychological Association [33], as well as the ethical recommendations for educational research [34], were followed in all phases of the study.

Approval of the research protocol was requested from the EDUCA Ethics Committee, which was approved under code 82019.

## 3. Results

### 3.1. Anthropometric Variables

#### 3.1.1. Descriptive and Functional Analysis

The paired-samples Student’s *t*-test was used to test whether there were statistically significant differences between the pre-lockdown and post-lockdown anthropometric parameters (Table 1). The results indicate that there are statistically significant differences (*p* < 0.05) in the average value of the variable between the two pre-lockdown measurements and the post-lockdown measurement, except for waist circumference (*p* = 0.690) where there is no statistically significant difference between the second pre-lockdown and post-lockdown data collection.

A paired-sample Student’s *t*-test was also performed to check whether there were statistically significant differences between pre-lockdown and post-lockdown anthropometric parameters by sex (Table 1). Again, there are statistically significant differences (*p* < 0.05) in the average value of the variable between the two pre-lockdown measurements and the post-lockdown measurement, with the exception of waist circumference in males (*p* = 0.0996) and in females (*p* = 0.592). Likewise, no statistically significant differences were observed in some anthropometric parameters between the two pre-lockdown measurements.

However, there were no statistically significant differences in the average value of anthropometric variables between men and women (*p* > 0.05), except for the subscapular skinfold in the second pre-lockdown data collection (*p* = 0.047).

#### 3.1.2. Evolution of Anthropometric Variables

**Figure 2:**
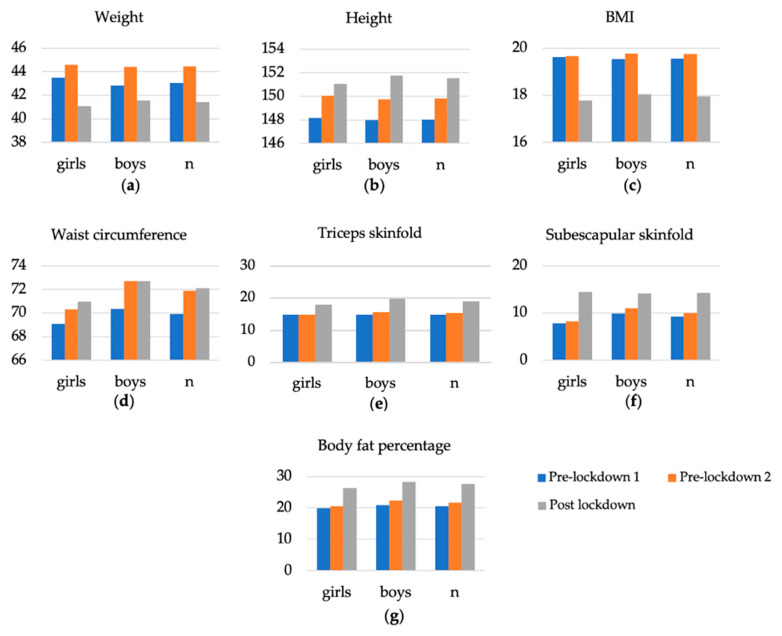
Changes in anthropometric variables in the sample and between genders. (**a**) Changes in the anthropometric variable of weight. (**b**) Changes in the anthropometric variable for height. (**c**) Changes in the anthropometric variable of BMI. (**d**) Changes in the anthropometric variable for waist circumference. (**e**) Trend of the anthropometric variable of triceps skinfold. (**f**) Evolution of the anthropometric variable of the subscapular skinfold. (**g**) Evolution of the anthropometric variable of the body fat percentage.

The results obtained for each of the anthropometric variables measured prior to lockdown varied slightly between the two data collections. However, there are significant differences between the results obtained for the different anthropometric variables in the pre-lockdown data collection compared to the data collection for the same variables post-lockdown. These differences can be seen in the total sample analyzed, as well as when the sample is broken down by gender (Table 2 and Figure 2).

The results indicate that between pre-lockdown two and post-lockdown there has been an increase in values above the standard values for height, weight and BMI corresponding to their age. These increases occurred in skinfold, waist circumference and body fat percentage measurements, while weight and BMI decreased in their values. Moreover, these changes were more accentuated in boys than in girls. This loss in weight and BMI values and the gain in fat mass could be due to the loss of muscle mass as a consequence of a deficit in physical activity [35].

#### 3.1.3. Relationships between Anthropometric Parameters

The results of the correlation tests between the different anthropometric variables were obtained using Pearson correlation test when the distribution of both variables is normal. Otherwise, the data were obtained by using the Spearman Rho correlation test.

The results obtained by Pearson correlation test were waist circumference in the first data collection with the same variable in the second data collection (r = 0.913), both pre-lockdown. The sub-scapular skinfold of the first data collection with the sub-scapular skinfold of the second data collection (r = 0.837), as well as with the post-lockdown measurement (r = 0.866), in the results obtained by Pearson correlation show a very high correlation (0.8 < r < 1).

In the case of the results of the correlation tests obtained by the Spearman Rho test between pre-lockdown one with pre-lockdown two and post-lockdown, the anthropometric parameters are presented in Table 3.

Table 4 presents the results of the relationships between pre-lockdown and post-lockdown anthropometric parameters.

The results of the relationship between pre-lockdown two and post-lockdown that show a very high correlation (0.8 < r < 1) focus on the anthropometric variables linked to body fat measurements in the research participants. BMI, waist circumference, triceps skinfold, subscapular skinfold and body fat percentage correlate very highly with each other. These correlation results show a positive increase of certain anthropometric parameters between pre-lockdown and post-lockdown values.

### 3.2. Dietary Habits

#### 3.2.1. Descriptive Analysis

In the analysis of the results on eating habits, data from 12 of the 42 items of the validated instrument for assessing food consumption, habits and practices in children aged 8–11 were used. Specifically, we selected those items that were particularly relevant in changing consumption habits: daily glasses of water, daily vegetable dishes or salads, daily pieces of fruit, daily bread, weekly amount of food rich in saturated fat, daily dairy products, weekly fish, daily glasses of soft drinks, weekly consumption of legumes, weekly consumption of sweets and jelly beans, weekly consumption of salty snacks and weekly consumption of cakes and sweet pastries, as well as two categorical items on the number of meals eaten per day and the type of food consumed in the mid-morning.

A descriptive and comparative analysis was made of the results obtained in the survey for the 12 items analyzed before and during lockdown, as well as for the two categorical items, type of food consumed mid-morning and number of meals per day. All the variables evaluated showed higher results between the type of food consumed and its frequency in pre-lockdown and during lockdown, except for dairy products consumed daily pre-lockdown (M = 2.48; SD = 0.93) compared to those consumed during lockdown (M = 2.20; SD = 0.92) and fish consumed weekly pre-lockdown (M = 1.70; SD = 0.90) compared to those consumed during lockdown (M = 1.60; SD = 0.94).

Likewise, to obtain the results of the categorical items, cross-tables were made between the frequency of food consumed pre-lockdown versus those consumed during lockdown, as well as the number of daily meals consumed. The results show an increase in the number of daily meals eaten during lockdown as compared to the pre-lockdown period, as well as an increase in the consumption of salty snacks (5.3%) and bread with additives (10%) and a decrease in the frequency of foods such as fruits consumed daily (−5.6%) during the lockdown period.

For both categorical items, Pearson chi-squared test of independence was performed, obtaining a significant result (*p* < 0.05). Therefore, with a confidence level of 95%, we can accept the hypothesis of dependence between the variables analyzed in the first categorical item (*p* < 0.030), as well as in the second one (*p* < 0.013).

Table 5 presents the results of the changes in feeding habits analyzed between pre-lockdown and post-lockdown.

#### 3.2.2. Significance Analysis

To check the normality of the variables, the Kolmogorov–Smirnov test was used, the result of this test being that the pre-lockdown and post-lockdown variables show significant results (*p* < 0.05), so we can accept the hypothesis that the variable does not have a normal distribution.

As the tests analyzed did not have a normal distribution, the Wilcoxon non-parametric test for related samples was performed. The results obtained are that there are statistically significant differences (*p* < 0.05) between three variables pre-lockdown with respect to post-lockdown in the average value of the variable. The variables are daily fruits (*p* < 0.048), daily dairy consumed (*p* < 0.038) and weekly salty snacks consumed (*p* < 0.021).

#### 3.2.3. Relationships between Dietary Habits

The post-lockdown anthropometric variables showed low (0.2 < r < 0.4) and very low (0 < r < 0.2) correlations with the pre-lockdown dietary variables as a whole. In these results, negative inverse correlation results also occur with low (−0.4 < r < −0.2) and very low (−0.2 < r < 0) results.

These same low and very low correlation results occur between the post-lockdown anthropometric variables and the dietary variables during lockdown. The correlations of height with daily glasses of water consumed (r < 0.427), which presents a moderate correlation (0.4 < r < 0.6), as well as the moderate negative or inverse correlations of weight with bread consumed daily (r = −0.400), triceps skinfold with sweets and treats consumed weekly (r = −0.476), stand out. Sweets and snacks consumed weekly also had a moderate negative or inverse correlation with sub-scapular skinfold (r = −0.489) and with body fat percentage (r = −0.486).

Finally, the results of correlation between the pre-lockdown feeding variables with those same variables during lockdown, present moderate correlation (0.4 < r < 0.6) between themselves, except for the glasses of water consumed daily (r = 0.685), the pieces of fruit consumed daily (r = 0.740), fish consumed per week (r = 0.703) and sweets and treats consumed per week (r = 0.642) are highly correlated (0.6 < r < 0.8).

### 3.3. Family Habits

#### 3.3.1. Descriptive Analysis

The family survey is made up of categorical variables in which a basic descriptive analysis has been carried out on the time spent by the sample on sedentary activities, such as exposure to screens, time spent on physical activity or time spent on other types of sedentary activities during the period of lockdown.

In relation to the time spent on screen exposure during the period of lockdown, 52% of the sample used the video-console for more than 60 min a day, 50% the television, 48% the computer, 30% the tablet and 26% the mobile phone for that same amount of time. In relation to educational and/or cultural activities, 82% of the sample spent more than 60 min on homework and 30% spent more than 146 min on homework. In the case of reading, 38% spent between 16 and 30 min per day, while 2% played musical instruments and 8% engaged in artistic activities for more than 60 min.

In terms of rest time measured by hours of sleep, 20% of the sample slept 8 h a day, 40% slept for 9 h and 38% slept for 10 h a day.

With regard to physical activity, there was a reversal in all the frequency ranges of physical activity evaluated. The percentage of children who were not physically active pre-lockdown (4%) increased during lockdown to 32% of the total sample, children who were physically active two or three times a week decreased by 10% during lockdown, between 4 and 5 days decreased by 14% and children who were physically active 6 to 7 days a week decreased from 10% pre-lockdown to 6% during lockdown.

Table 6 presents the results of changes in physical activity habits analyzed between pre-lockdown and during lockdown.

In addition, also noteworthy are the differences in the sample results according to the place of residence and the type of dwelling in which they lived during the lockdown. Fifty-six percent of the sample resided in an urban setting, 38% in a semi-urban or residential setting and 6% in a rural setting. The participants who showed higher values for anthropometric parameters were those who resided in urban versus semi-urban or residential settings and with more significant differences compared to those who resided in rural settings.

Regarding the size of the dwelling in which the children resided during the lockdown, 8% resided in a flat of less than 60 m^2^, 28% in a flat between 61 and 90 m^2^, 28% in a flat between 91 and 120 m^2^ and 36% of the sample resided in a house with a garden. Higher results in anthropometric parameters were obtained for children living in smaller dwellings compared to larger dwellings with a garden.

#### 3.3.2. Statistically Significant Differences

To test for significant differences between the anthropometric variables and the variables collected by means of the family survey, the Mann–Whitney U test and the Kruskal–Wallis H test were used. According to these tests of independence, there is no statistically significant difference in the mean value of the variable between the different groups of variables (*p* > 0.05). Exceptions occur in the anthropometric variable of post-lockdown height with the variable of physical activity practice of the child pre-lockdown (*p* = 0.033) and in the variable of post-lockdown weight with the variable of average daily hours of sleep of the child (*p* = 0.045).

#### 3.3.3. Post-Lockdown Results

The results obtained from the anthropometric variables post-lockdown in relation to the variables of the family habits analyzed on the physical activity of the child and other sedentary activities carried out during lockdown have been extracted.

The most significant result of the anthropometric variables during lockdown in relation to the frequency of physical activity (Table 7) is the increase in anthropometric values in children who did not practice physical activity before or after lockdown compared to children who did practice physical activity and thus have maintained more stable values of anthropometric parameters.

The results obtained for the different anthropometric variables analyzed during lockdown were also extracted in relation to the time the sample spent on activities related to exposure to screens during the lockdown period (Table 8). Children who spent a greater number of minutes on screens during lockdown were those whose anthropometric parameter values were more altered than those who did not spend minutes on this type of sedentary activity, such as the use of tablets or computers, and especially the use of video consoles, television and mobile phones.

Finally, the results obtained for the different anthropometric variables analyzed during lockdown were extracted in relation to the time the sample spent on other sedentary activities during the period of lockdown, such as schoolwork, reading, playing musical instruments or artistic activities (Table 9). These results show changes in anthropometric values in children who spent more minutes per day in these types of activities.

## 4. Discussion

The aim of this study was to determine the impact that lockdown due to SARS-CoV-2 had on anthropometric parameters in children aged 11/12.

This study showed results between pre-lockdown and post-lockdown that show changes in anthropometric values outside the expected standardized values for height, weight and BMI [26,31]. The results show a decrease in BMI and body weight and an increase in waist circumference, body fat percentage and skinfold parameters. Likewise, this research has shown that the habits in relation to physical activity and eating habits were modified by the sample during the period of lockdown. In relation to physical activity habits, there was a decrease in the number of minutes per day, as well as in the weekly frequency of physical activity. In terms of dietary habits, the number of meals per day increased, as well as the intake of foods rich in saturated fat and sugars. The results show worse values for anthropometric parameters in boys than in girls, as well as in study participants who lived in small dwellings without a garden during confinement and in children whose parents were less educated.

Furthermore, different studies show that children and adolescents do not comply with the recommendations regarding physical activity and sedentary behavior [36], which together with the changes in habits brought about by the health crisis created by COVID-19 [4,37,38] may lead to increases in anthropometric parameters and accentuate problems of overweight and obesity. The percentages of children who exceed the minutes of screen time recommended by the WHO [16] are high and together with the time spent in other sedentary activities, favor an increase in the rate of overweight and obesity. Finally, the number of hours of sleep per day is also an important factor favoring changes in anthropometric parameters [39,40]. The WHO recommendation for hours of sleep per day in children aged 6 to 12 years is at least 11 h.

This research also shows that the changes in habits produced by the lockdown due to SARS-CoV-2 [4,37,38,41] have had an influence on eating habits, leading to a greater number of meals per day, as well as an increase in the intake of foods rich in saturated fats and sugars, which also favors an increase in anthropometric parameters and the appearance of childhood overweight and obesity [42,43].

This research also confirms that the time dedicated to physical activity during lockdown does not reach the minimum time recommended by the WHO [18,44], which has also favored changes in anthropometric parameters, as shown in the results obtained for anthropometric parameters in relation to the time and frequency of physical activity before and after lockdown.

Therefore, this research confirms that environmental variables were the main causes of anthropometric changes in the study sample. Numerous research studies and institutions have found that environmental variables are the main factors in the increase of obesity and overweight rates in healthy populations [45,46,47].

This study shows that anthropometric parameters according to reference values [29,31] have been modified between pre-lockdown and post-lockdown due to SARS-CoV-2, because of a multifactorial cause. These changes in anthropometric parameters are more accentuated in the case of boys, with the value of some parameters being high. However, the results obtained do not show any evidence of childhood obesity problems in the sample [48]. It should be noted that, after lockdown, the anthropometric parameters of weight and the BMI of the sample decreased, while the parameters of skinfolds and body fat percentage increased. These changes are due to the long period of physical inactivity or the lack of physical stimuli produced in the body during lockdown [49,50,51]. Tests performed to verify the relationships between anthropometric variables have shown very high correlations between pre-lockdown two and post-lockdown in the anthropometric variables of waist circumference, in the different measurements of subcutaneous folds and in the percentage of body fat. This increase in body fat disproportionate to the normal growth of the child may be due to the impact of environmental variables on the modification of anthropometric parameters during SARS-CoV-2 lockdown [52,53].

Until the outbreak of SARS-CoV-2, different reports and organizations had already noted the high levels of childhood overweight and obesity in Spain, i.e., 23.3% and 17.3%, respectively [54,55], and their association with other pathologies, such as cardiovascular diseases, metabolic diseases, cancer or hypertension, among others, which can manifest themselves in childhood and adolescence or appear in adulthood [56,57,58,59]. These diseases associated with childhood overweight and obesity may become risk factors in the event of SARS-CoV-2 infection [13,41,60,61]. The large Spanish lockdown may have favored the increase of these values of childhood overweight and obesity.

## 5. Conclusions

Based on the results obtained in this research, it is concluded that there have been significant changes in the anthropometric parameters of 11- and 12-year-old boys and girls in an educational Centre in Cantabria (Spain) as a result of the impact of the lockdown due to SARS-CoV-2. These changes may have had a multifactorial origin in which an excess of screen time, changes in eating habits and a decrease in physical activity time during lockdown have predominated. In relation to gender, these changes have been more significant in boys than in girls. However, it should be noted that one of the limitations of the research is the difference in sample size by gender. Another limitation of the study is the size of the sample, which is why it is not possible to generalize the results of the study to the population aged 6–12 years. However, it should be noted that, taking into account the aforementioned studies, as well as the results of the latest survey of the Spanish National Institute of Statistics (INE) in 2020 [62] used for the elaboration of the EUROSTAT reports [63], a similar prevalence is observed between the convenience sample of this study and the general population in relation to the rates of overweight and obesity, the values of physical activity practice and eating habits. Despite the fact that all anthropometric parameters have increased, values of childhood obesity have not been reached.

This research has shown the need to continue strengthening and promoting effective strategies to encourage the practice of healthy and regular physical activity, the acquisition and maintenance of healthy eating habits and the responsible use of technology, especially in situations such as the large-scale lockdown due to SARS-CoV-2. There is an urgent need to design and implement a strategic plan due to the negative consequences that the lockdown has had on the child and adolescent population in terms of anthropometric parameters. It would be desirable for the health authorities, educational institutions and the family environment to participate in this plan.

## Figures and Tables

**Table 1:** Significance results for anthropometric variables as a function of data collection and gender.

	Pre-Lockdown 1–Pre-Lockdown 2			Pre-Lockdown 1–Post-Lockdown			Pre-Lockdown 2–Post-Lockdown		
	*n* (Total)	Boys	Girls	*n* (Total)	Boys	Girls	*n* (Total)	Boys	Girls
W	0.000	0.000	0.000	0.030	0.134	0.129	0.000	0.001	0.041
H	0.000	0.000	0.000	0.000	0.000	0.000	0.000	0.000	0.044
BMI	0.045	0.022	0.731	0.000	0.000	0.025	0.000	0.000	0.028
WC	0.000	0.001	0.124	0.000	0.005	0.024	0.690	0.996	0.592
TS	0.044	0.010	0.952	0.000	0.000	0.000	0.000	0.000	0.000
SS	0.002	0.005	0.226	0.000	0.001	0.007	0.000	0.000	0.006
% BF	0.001	0.001	0.293	0.000	0.000	0.000	0.000	0.000	0.001

Note: *n* = 50; boys = 33; girls = 17. W, weight; H, height; BMI, body mass index; WC, waist circumference; TS, triceps skinfold; SS, subscapular skinfold; %BF, body fat percentage.

**Table 2:** Results of anthropometric variables as a function of data collection and gender.

	Pre-Lockdown 1			Pre-Lockdown 2			Post-Lockdown		
	*n* (Total)	Boys	Girls	*n* (Total)	Boys	Girls	*n* (Total)	Boys	Girls
W	43.05 ± 8.72	42.84 ± 7.43	43.47 ± 11.05	44.47 ± 9.01	44.40 ± 7.66	44.59± 11.46	41.40 ± 11.19	41.57 ± 9.63	41.08 ± 14.06
H	148.03 ± 6.85	147.96 ± 5.60	148.17 ± 9.00	149.82 ± 6.96	149.72 ± 5.63	150.02 ± 9.21	151.52 ± 7.00	151.75 ± 6.07	151.05 ± 8.72
BMI	19.56 ± 3.26	19.53 ± 3.00	19.61 ± 3.82	19.74 ± 3.44	19.77 ± 3.08	19.66 ± 4.17	17.95 ± 4.39	18.04 ± 4.11	17.77 ± 5.03
WC	69.91 ± 7.76	70.33 ± 7.05	69.08 ± 9.15	71.89 ± 8.91	72.70 ± 8.57	70.32 ± 9.60	72.11 ± 9.40	72.71 ± 8.83	70.94 ± 10.60
TS	14.76 ± 6.42	14.71 ± 6.43	14.85 ± 6.59	15.30 ± 6.55	15.54 ± 6.78	14.82 ± 6.25	19.08 ± 8.49	19.68 ± 8.88	17.91 ± 7.79
SS	9.18 ± 4.99	9.87 ± 5.83	7.82 ± 2.35	10.02 ± 5.50	10.92 ± 6.29	8.26 ± 2.92	14.29 ± 10.35	14.16 ± 10.43	14.52 ± 10.49
%BF	20.58 ± 8.56	20.89 ± 9.65	19.99 ± 6.12	21.73 ± 8.91	22.36 ± 10.20	20.51 ± 5.72	27.63 ± 13.41	28.27 ± 14.74	26.38 ± 10.68

Note: Data are presented as average ± standard deviation; n = 50; boys = 33; girls = 17. W, weight; H, height; BMI, body mass index; WC, waist circumference; TS, triceps skinfold; SS, subscapular skinfold; %BF, body fat percentage.

**Table 3:** Correlation results of pre-confinement 1, pre-confinement 2 and post-confinement anthropometric parameters.

Pre-Lockdown 1	Pre-Lockdown 2							Post-Lockdown						
	W	H	BMI	WC	TS	SS	%BF	W	H	BMI	WC	TS	SS	%BF
W	0.984	0.515	0.875	0.842	0.698	0.712	0.678	0.849	0.508	0.773	0.876	0.626	0.726	0.683
H	0.585	0.975	0.192	0.268	0.016	0.092	0.794	0.473	0.962	0.212	0.354	−0.055	0.084	0.001
BMI	0.863	0.114	0.985	0.889	0.763	0.825	0.822	0.773	0.111	0.823	0.861	0.814	0.853	0.851
WC	0.885	0.234	0.934	-	0.699	0.787	0.764	0.821	0.233	0.830	0.935	0.775	0.817	0.815
TS	0.628	−0.055	0.801	0.732	0.944	0.836	0.936	0.599	−0.054	0.690	0.685	0.890	0.835	0.896
SS	0.749	0.038	0.886	-	0.843	-	0.917	0.733	0.030	0.791	0.772	0.861	0.928	0.918
%BF	0.687	−0.014	0.834	0.767	0.925	0.888	0.951	0.648	−0.018	0.724	0.712	0.895	0.874	0.919

Note: Data are presented as correlation coefficient. W, weight; H, height; BMI, body mass index; WC, waist circumference; TS, triceps skinfold; SS, subscapular skinfold; %BF, body fat percentage.

**Table 4:** Correlation results of pre-confinement and post-confinement anthropometric parameters.

Pre-Lockdown 2	Post-Lockdown						
	W	H	BMI	WC	TS	SS	%BF
W	0.859	0.518	0.771	0.873	0.621	0.723	0.674
H	0.420	0.975	0.144	0.287	−0.128	0.010	−0.070
BMI	0.777	0.131	0.816	0.871	0.808	0.842	0.838
WC	0.822	0.176	0.843	0.903	0.807	0.831	0.835
TS	0.578	−0.042	0.648	0.684	0.901	0.825	0.893
SS	0.733	0.021	0.793	0.761	0.849	0.911	0.901
%BF	0.652	−0.039	0.730	0.733	0.909	0.901	0.936

Note: Data are presented as correlation coefficient. W, weight; H, height; BMI, body mass index; WC, waist circumference; TS, triceps skinfold; SS, subscapular skinfold; %BF, body fat percentage.

**Table 5:** Results of changes in feeding habits pre-lockdown and post-lockdown.

	Pre-Lockdown	Post-Lockdown
Glasses of water/day	4.38 ± 1.70	4.50 ± 1.81
Vegetable dishes or salad/day	1.46 ± 0.97	1.60 ± 0.96
Fruit/day	2.62 ± 1.19	2.92 ± 1.42
Bread/day	2.54 ± 2.12	3.00 ± 2.24
High saturated fat food/week	1.50 ± 0.95	1.74 ± 1.00
Dairy/day	2.48 ± 0.93	2.20 ± 0.92
Fish/weekly	1.70 ± 0.90	1.60 ± 0.94
Glasses of soft drinks/day	1.10 ± 1.31	1.44 ± 1.76
Pulses/weekly	2.16 ± 1.21	1.96 ± 0.85
Sweets and sweets/weekly	2.04 ± 1.47	2.14 ± 1.29
Savory snacks/weekly	1.44 ± 0.83	1.78 ± 1.26
Cakes and pastries/weekly	0.90 ± 0.81	1.04 ± 0.88

Note: Data are presented as mean ± standard deviation.

**Table 6:** Results of changes in physical activity habits pre-lockdown and during lockdown.

	Pre-Lockdown			During-Lockdown		
	*n*	%	A%	*n*	%	A%
No physical exercise	2	4%	4%	16	32%	32%
2 or 3 times/week	23	46%	50%	18	36%	68%
4 or 5 times/week	20	40%	90%	13	26%	94%
6 or 7 times/week	5	10%	100%	3	6%	100%
TOTAL	50	100%		50	100%	

**Table 7:** Results of pre- and post-lockdown anthropometric variables in relation to the frequency of children who did not practice physical activity.

PA	*n*	W	H	BMI	WC	TS	SS	%BF
PL	2	30.00 ± 5.79	145.00 ± 1.41	14.29 ± 3.03	72.00 ± 16.26	20.00 ± 11.31	12.00 ± 8.48	26.02 ±12.43
DL	16	39.47 ± 9.15	149.30 ± 7.94	17.67 ± 3.60	71.55 ± 9.74	20.40 ± 9.03	16.35 ± 10.81	30.18 ± 14.88

Note: Data are presented as average ± standard deviation. PA, physical activity; PL, pre-lockdown; DL, during lockdown; *n*, sample cases; W, weight; H, height; BMI, body mass index; WC, waist circumference; TS, triceps skinfold; SS, subscapular skinfold; %BF, body fat percentage.

**Table 8:** Results of post-lockdown anthropometric variables in relation to daily time spent in activities related to screen exposure.

Act.	Time min/Day	*n*	W	H	BMI	WC	TS	SS	%BF
GC	0	16	37.98 ± 11.76	150.87 ± 8.92	16.63 ± 4.70	67.37 ± 7.28	15.93 ± 6.34	11.43 ± 8.23	23.08 ± 9.12
	76–100	6	43.14 ± 8.37	153.06 ± 6.56	18.37 ± 3.79	74.80 ± 8.45	21.70 ± 7.68	16.33 ± 10.74	31.33 ± 13.80
TV	0	4	38.40 ± 15.18	145.25 ± 8.77	17.76 ± 4.89	65.12 ± 6.70	17.87 ± 3.19	13.37 ± 7.47	25.95 ± 6.64
	76–100	2	42.45 ± 23.40	149.00 ± 4.24	18.84 ± 9.46	75.00 ± 20.50	16.50 ± 6.36	13.00 ± 9.89	24.66 ± 10.50
PC	0	10	42.64 ± 13.76	150.60 ± 7.44	18.63 ± 5.24	71.85 ± 13.45	20.70 ± 10.77	15.75 ± 12.98	30.31 ± 18.01
	76–100	4	42.87 ± 18.93	157.25 ± 6.94	17.21 ± 6.81	72.37 ± 11.07	17.50 ± 12.36	15.50 ± 17.05	24.97 ± 17.97
TB	0	17	46.26 ± 11.64	154.17 ± 7.38	19.45 ± 4.70	75.11 ± 9.70	20.11 ± 8.99	15.58 ± 11.24	29.43 ± 13.85
	76–100	2	38.75 ± 18.17	147.50 ± 2.12	17.69 ± 7.84	70.75 ± 14.49	18.50 ± 9.19	13.25 ± 10.25	27.28 ± 14.21
MP	0	26	39.99 ± 10.52	150.38 ± 7.84	17.56 ± 3.92	70.61 ± 8.66	17.92 ± 7.18	12.42 ± 7.96	25.56 ± 11.48
	116–130	6	51.01 ± 12.67	155.50 ± 6.02	21.11 ± 5.22	76.66 ± 11.60	23.00 ± 2.53	21.83 ± 16.26	35.58 ± 19.58

Note: Data are presented as average ± standard deviation; *n*, sample cases; Act., activity; GC, game console; TV, television; PC, personal computer; TB, tablet; MP, mobile phone; W, weight; H, height; BMI, body mass index; WC, waist circumference; TS, triceps skinfold; SS, subscapular skinfold; %BF, body fat percentage.

**Table 9:** Results of post-lockdown anthropometric variables in relation to daily time spent in other sedentary activities.

Act.	Time min/Day	*n*	W	H	BMI	WC	TS	SS	%BF
SW	31–45	1	39.90 ± 0	151.00 ± 0	17.49 ± 0	73.50 ± 0	22.00 ± 0	16.00 ± 0	31.45 ± 0
	76–100	5	45.00 ± 6.53	152.60 ± 6.06	19.34 ± 2.94	74.80 ± 7.91	19.60 ± 10.15	15.40 ± 12.13	29.18 ± 17.37
R	0	8	45.10 ± 12.48	150.12 ± 11.08	19.68 ± 2.92	73.62 ± 9.39	20.25 ± 8.87	16.56 ± 10.91	29.34 ± 11.41
	31–45	2	47.45 ± 17.18	145.00 ± 1.41	22.49 ± 7.73	77.25 ± 15.20	25.50 ± 11.31	15.50 ± 10.60	34.09 ± 16.75
MI	0	45	41.46 ± 11.53	151.60 ± 7.08	17.95 ± 4.50	72.18 ± 9.75	19.07 ± 8.76	14.78 ± 10.72	27.98 ± 13.90
	76–100	1	43.30 ± 0	148.00 ± 0	19.76 ± 0	65.50 ± 0	22.00 ± 0	10.00 ± 0	27.13 ± 0
AA	0	19	45.11 ± 11.87	151.52 ± 7.61	19.46 ± 4.15	75.18 ± 10.67	20.89 ± 9.60	16.92 ± 11.04	30.75 ± 14.70
	76–100	1	49.30 ± 0	160.00 ± 0	19.25 ± 0	80.50 ± 0	22.50 ± 0	8.00 ± 0	26.06 ± 0

Note: Data are presented as average ± standard deviation; *n*, sample cases; SW, school work; R, reading; MI, playing musical instruments; AA, artistic activities; W, weight; H, height; BMI, body mass index; WC, waist circumference; TS, triceps skinfold; SS, subscapular skinfold; %BF, body fat percentage.

## Data Availability

Data sharing is not applicable.
